# Automated stress detection using mobile application and wearable sensors improves symptoms of mental health disorders in military personnel

**DOI:** 10.3389/fdgth.2022.919626

**Published:** 2022-08-23

**Authors:** Brent D. Winslow, Rebecca Kwasinski, Jeffrey Hullfish, Mitchell Ruble, Adam Lynch, Timothy Rogers, Debra Nofziger, William Brim, Craig Woodworth

**Affiliations:** ^1^ Design Interactive, Inc., Orlando, FL, United States; ^2^ Department of Medical and Clinical Psychology, Center for Deployment Psychology, Uniformed Services University of the Health Sciences, Bethesda, MD, United States; ^3^ Department of Behavioral Health, Brook Army Medical Center, Fort Sam Houston, TX, United States

**Keywords:** stress, telemedicine, mental health, cognitive behavioral therapy (CBT), wearable technology, mobile applications, sleep

## Abstract

Leading causes in global health-related burden include stress, depression, anger, fatigue, insomnia, substance abuse, and increased suicidality. While all individuals are at risk, certain career fields such as military service are at an elevated risk. Cognitive behavioral therapy (CBT) is highly effective at treating mental health disorders but suffers from low compliance and high dropout rates in military environments. The current study conducted a randomized controlled trial with military personnel to assess outcomes for an asymptomatic group (*n* = 10) not receiving mental health treatment, a symptomatic group (*n* = 10) using a mHealth application capable of monitoring physiological stress *via* a commercial wearable alerting users to the presence of stress, guiding them through stress reduction techniques, and communicating information to providers, and a symptomatic control group (*n* = 10) of military personnel undergoing CBT. Fifty percent of symptomatic controls dropped out of CBT early and the group maintained baseline symptoms. In contrast, those who used the mHealth application completed therapy and showed a significant reduction in symptoms of depression, anxiety, stress, and anger. The results from this study demonstrate the feasibility of pairing data-driven mobile applications with CBT in vulnerable populations, leading to an improvement in therapy compliance and a reduction in symptoms compared to CBT treatment alone. Future work is focused on the inclusion of passive sensing modalities and the integration of additional data sources to provide better insights and inform clinical decisions to improve personalized support.

## Introduction

Mental health disorders, including major depressive disorder (MDD), generalized anxiety disorder (GAD), and post-traumatic stress disorder (PTSD) represent leading causes in global health-related burden (1), and have been significantly exacerbated by the COVID-19 pandemic (2). Such disorders present with a variety of symptoms, including stress, depression, anger, fatigue, insomnia, substance abuse, and increased suicidality (3, 4). Available evidence suggests that particular professions have an increased risk for symptoms of mental health disorders, including first responders (5), medical professionals (6), and members of the armed forces. Within the latter, the literature increasingly supports a Consequence of War Syndrome (CWS) to describe a cluster of symptoms experienced by members of the armed forces including chronic pain, insomnia, and other physical complaints along with PTSD, anxiety, depression, anger, and neuropsychological deficits (7). Unlike previous symptom clusters such as Gulf War Syndrome (8), CWS appears to be fundamentally linked to chronic stressors inherent to military deployment (7). Given the increasing prevalence of mental health disorders, there is an increasing need for support tools.

Cognitive behavioral therapy (CBT) has emerged as one of the most effective psychotherapy modalities used to treat a range of emotional and physiological symptoms such as depression, anxiety, stress, anger, PTSD, and insomnia (9). CBT is generally administered by mental health professionals, and consists of a structured, collaborative process that helps individuals consider and alter their thought processes and behaviors, usually administered weekly over several months. However, while CBT has been shown to be highly effective in the general population, efficacy in military populations has been limited (10), in which CBT is associated with low compliance, and high drop-out rates (11). Additional challenges to therapy provision in the military health system include the fact that many service members choose not to seek care (12), experience long wait times (13), and participate at low rates in clinical studies (14). Within CBT, much of the improvement in symptoms occurs due to skill practice *via* homework assignments (15), but homework compliance in CBT remains problematic. In addition, standard CBT does not offer the provider objective or verifiable information regarding the utilization of therapeutic skills outside of office visits (16), including use of the relaxation and behavioral strategies shown to be the most effective component of treatment (17).

Previous work by our group has indicated that mHealth technology capable of monitoring and alerting participants to stress, anger, and anxiety triggers, while offering such information to providers, is capable of increasing CBT efficacy in a small randomized controlled trial (RCT) using a military veteran population (18). Other groups have highlighted the need for promoting engagement with mobile applications to maximize efficacy (19, 20). There is a need to apply similar technology in active duty military populations, closer to the point at which symptoms develop. In the current study, the ability of CBT to reduce symptoms of stress, anger, and associated effects on other behaviors (e.g., sleep) with and without an mHealth application was evaluated with 20 active duty service members reporting stress or anger who were undergoing CBT, compared to 10 asymptomatic active duty service members. The primary objective was to measure the effectiveness of the mHealth application in tandem with CBT to reduce anger and stress in active duty service members. We hypothesize that use of the mHealth application will result in significantly less anger, anxiety, depression, and PTSD symptoms following CBT treatment as compared to standard CBT therapy in an active duty population.

## Materials and methods

### Participants

Thirty participants (ten per group) were recruited for this study based on a power analysis using data from prior work (*d* = 1.0, *α* = 0.05, *β* = 0. 8, Wilcoxon–Mann–Whitney test) (18). Participants included both symptomatic and asymptomatic active duty military personnel, aged 18–64. Symptomatic participants were recruited at Brooke Army Medical Center (BAMC, Ft. Sam Houston, TX, United States) from active duty military personnel who presented to the nonemergency, outpatient mental health clinic. Potential participants were processed through the standard clinic intake or triage protocol. Clinic providers were briefed on the study availability and inclusion criteria. Participants that met the initial inclusion criteria (diagnosis or complaint of stress and anger), were offered to schedule with a project coordinator. The project coordinator explained the protocol and completed the consent if the participant was appropriate and volunteered for inclusion. Asymptomatic participants were recruited among active duty military personnel in Orlando FL using word of mouth.

### Experimental procedure

The study design was a parallel, RCT with active duty populations to test the effectiveness of a novel mHealth application in conjunction with CBT (experimental group) compared to CBT alone (control group), and to an additional psychologically asymptomatic control group (asymptomatic group) that used the mHealth application. Symptomatic participants were randomly assigned to a treatment group *via* block randomization to ensure equal and random groups. Asymptomatic group participants were not randomized.

After obtaining written informed consent, participants were given a unique four-digit study participant ID number and were asked to complete baseline self-report questionnaires (see Section “Self-reported measures”). Ten active duty service members were randomized to receive the mHealth mobile application as part of their CBT (experimental group), and ten were randomized to receive standard CBT without the mHealth application (control group). An additional ten active duty service members that were not reporting stress or anger, (asymptomatic group) received a smartwatch and mHealth mobile application to determine the ability of the system to detect anger and stress in a healthy cohort and to compare any changes that occur from using the mHealth application alone to CBT with the mHealth application in a symptomatic population. After random assignment to treatment condition, participants engaged in the protocol treatment with a study therapist. Individuals not interested or deemed not appropriate for the study, were scheduled for a routine follow up appointment consistent with the clinic policy. Participants randomized to the experimental group received a smartwatch along with instructions in its use, and downloaded the mHealth application to their Android or iOS phone (see Section “Physiological measures”). To control for possible effects of using study equipment, participants randomized to the control group received a smartwatch only and instructions in its use but were not provided with the download of the mHealth application.

All symptomatic participants received standard CBT consistent with the U.S. Department of Health and Human Services Substance Abuse and Mental Health Services Administration (SAMHSA) CBT therapy manual targeting stress and anger management, in a one-on-one setting (21). The interventions used in this study are manualized treatments widely used in both civilian and military/veteran populations and represent the current standard of care. The expected treatment course is approximately eight to twelve sessions, approximately 2–3 months for weekly sessions. All participants completed the study measures at baseline and every 4th session to completion of treatment. Prior to appointments with participants assigned to the experimental group, the study therapist accessed the participant's physiological data (times and locations of situations that are associated with stress/anger events and any journal entries) *via* a secure cloud server and a password-protected, HIPAA-compliant provider portal. The mHealth data since the last appointment was discussed in the course of the treatment session as an adjunct to the standard CBT protocol; as such, therapists were not blinded to experimental condition.

The study terminated after the 12th scheduled appointment although participants were allowed to continue with the study therapist or referred to another treating provider if continued treatment was indicated. Participants were considered a dropout if they left treatment after having completed the first appointment. Participants were considered completed at the 12th session or if the participant and therapist agreed that clinical goals were met at any point prior to the 12th session. Follow-up analyses were assessed for the participant's final session whether completed or considered an early drop-out. As such, all analyses described within and between participants at baseline and follow-up included groups of ten. Participants who used the mHealth application also completed a mobile application questionnaire at the final appointment.

Asymptomatic controls were recruited from other sites through the use of fliers. Interested participants met with a project coordinator and, if they met inclusion criteria, were consented into the study. Asymptomatic participants completed the same behavioral health assessment measures and received a smartwatch and the mHealth mobile application along with instructions in its use. Data from the asymptomatic controls was uploaded from the participant phone to the secure cloud server.

### Self-reported measures

Participants provided demographics information at their initial appointment. The Depression, Anxiety, Stress Scale (DASS) (22), Patient-reported outcomes measurement information scale (PROMIS)-Anger Scale (23), PTSD Checklist for DSM-5 (PCL-5) (24), and Epworth Sleepiness Scale (ESS) (25) were also provided at the initial appointment, and every 4th session (up to 12 sessions) following the start of the study as part of CBT.

### Physiological measures

In previous work, the integration of data from a research-grade wrist-worn wearable [Empatica E3; (26)] paired with an Android mHealth application that objectively identifies stress and prompts participants to engage in cognitive and behavioral skills taught in CBT was shown to significantly improve adherence with CBT and also resulted in significant reductions to stress, anger, and anxiety in a group of military veterans compared to CBT alone (18). In the current study, the mHealth application was implemented in both Android and iOS and provided to participants in the experimental and asymptomatic groups for download on their personal device. The mHealth application received raw sensor data from a series of commercial smartwatches (Garmin, Olathe KS) including the fēnix® 5, fēnix® 6, and vívoactive® 4 series. Participants provided a 5 min baseline recording, following which data from the smartwatch was classified using a cardiovascular algorithm of stress developed previously (18) which alerted users through the mHealth application when physiological stress was identified. The stress algorithm used pulse plethysmography (PPG) to derive frequency and temporal domain metrics of heart rate variability (27) and respiration rate, along with an embedded inertial measurement unit (IMU) to give context to the raw data. The algorithm is 97.1% accurate in capturing stress, and is scaled from 1 to 10, with 1 to 3 representing low stress, 4 to 7 representing moderate stress, and 8 to 10 representing severe stress (18). When the algorithm identified stress, participants were asked to provide self-reported information regarding the stressor including the stress level and trigger, and were prompted to perform guided stress and anger reduction techniques, including deep breathing exercises (28), progressive muscle relaxation (29), biofeedback (30), and meditation (31) available in the application. The application leveraged techniques designed to promote user engagement, including interactive elements (e.g., biofeedback), educational materials, homework, and support tools (32). The mHealth application also presented daily, weekly, or monthly stress events and sleep metrics including sleep fragmentation, defined as the number of minutes awake divided by the total minutes between falling asleep and awakening. The mHealth application also provided resources for emergency services, contact lists for support, and educational information on the effects of stress and fatigue. The application also provided journaling capabilities to support CBT. A web-based provider portal was implemented *via* a secure cloud server and allowed CBT providers to view physiological data for individual participants, utilization of skills being used in therapy, and enter reminders which were sent to the mobile application.

### Data analysis and statistics

Mixed model ANOVA analyses were used to analyze differences between groups with a within group factor of timepoint and a between group factor of condition (experimental, control, asymptomatic). Effect size was calculated using eta squared (*η*^2^), the ratio of the sum of squares between groups to the total sum of squares. The guidelines for interpreting *η*^2^ are: 0.01 = small effect; 0.06 = moderate effect; 0.14 = large effect (33). Within groups, paired *t*-tests were used to establish differences between the initial and final session. All statistical testing was done in SPSS software.

## Results

The sociodemographic factors in the evaluation are listed in (Table 1). The average age of the participants was 37.4 ± 7.7 (SD) years, most were male, and most were in the Army.

**Table 1:** List of sociodemographic factors of study sample.

	Study sample % (*n*)
**Gender**	
Male	80.0 (24)
Female	20.0 (6)
**Age group**	
20–29	16.7 (5)
30–39	43.3 (13)
40–49	33.3 (10)
50–59	6.7 (2)
**Military branch**	
Army	53.3 (16)
Navy	13.3 (4)
Air Force	10.0 (3)
Marines	23.3 (7)

Among the symptomatic groups receiving CBT, five individuals dropped out prior to completion of therapy, all of which had been randomized to the control group. Independent samples *t*-test indicated that individuals in the symptomatic experimental group completed a significantly greater number of therapy sessions (*p* = 0.028) at an average of 9.2 ± 2.8 (SD) sessions as compared to 5.6 ± 3.9 (SD) in the symptomatic control group. The effect size, calculated using eta squared (*η*^2^), was large at 0.24. The asymptomatic control group was assessed at 12 weeks following commencement of the study.

Depression, anxiety, and stress scale (DASS) scores are shown in Table 2. For the initial assessment (baseline), the asymptomatic group was considered normal, while the control group reported depression in the 83rd percentile (mild), with anxiety and stress in the 96th percentile (severe) as compared to a normative sample (34). The experimental group reported depression in the 86th percentile (mild), anxiety in the 95th percentile (severe), and stress in the 91st percentile (moderate) (22).

**Table 2:** Mean (SD) DASS assessment scores.

DASS scale	Initial assessment			Follow-up		
	Depression	Anxiety	Stress	Depression	Anxiety	Stress
Asymptomatic (*n* = 10)	3.1 (3.5)	1.6 (3.1)	7.3 (3.5)	2.5 (3.8)	2.2 (2.4)	9.7 (7.6)
Control (*n* = 10)	10.8 (8.9)	17.2 (9.4)	29.8 (7.1)	11.4 (8.7)	12.0 (8.7)	24.2 (11.7)
Experimental (*n* = 10)	11.6 (5.8)	14.2 (12.4)	21.0 (9.8)	5.0 (5.0)^*^	5.0 (5.0)^*^	14.2 (6.0)^*^

* Indicates significant differences within groups at *p* ≤ 0.05.

A one-way between-groups analysis of variance (ANOVA) was used to assess differences in self-reported depression, anxiety, and stress (DASS) between groups at baseline. For depression, there was a statistically significant difference at baseline with a large effect size [*F*(2,27) = 5.29, *p* = 0.011, *η*^2^ = 0.28]. *Post-hoc* comparisons indicated that the asymptomatic group reported a significantly lower depression score than the symptomatic control group (*p* = 0.033) and symptomatic experimental group (*p* = 0.017). No differences were observed between the two symptomatic groups (*p* = 0.958). There was also a statistically significant difference in self-reported anxiety with a large effect size [*F*(2,27) = 8.19, *p* = 0.002, *η*^2^ = 0.38]. *Post-hoc* comparisons indicated that the asymptomatic group reported significantly less anxiety than the symptomatic control group (*p *= 0.002) and symptomatic experimental group (*p* = 0.013). No differences were observed between the two symptomatic groups (*p* = 0.748). Finally, there was a statistically significant difference in stress scores with a large effect size [*F*(2,27) = 24.21, *p* < 0.001, *η*^2^ = 0.64]. *Post-hoc* comparisons indicated that the mean for the asymptomatic group was significantly lower than the symptomatic control group (*p* < 0.001) and symptomatic experimental group (*p* = 0.001). No difference was observed between the symptomatic groups (*p* = 0.300).

Depression, anxiety, and stress scale (DASS) scores during the follow-up are also shown in Table 2. At follow-up the asymptomatic group retained normal levels of depression, anxiety, and stress, while the symptomatic control group reported mild depression, with stress and anxiety in the 94th percentile (moderate). In contrast, the symptomatic experimental group reported depression, anxiety, and stress at normal levels (22). At the follow-up assessment, the symptomatic experimental group reported levels of stress, depression, and anxiety that did not differ statistically from the asymptomatic group as described below, indicative of successful therapy and reduction of symptoms. However, the symptomatic control group reported values during their final follow-up were not statistically different from the initial assessment as described below, indicative of a lack of therapeutic progress. There was a significant difference between groups in self-reported depression with a large effect size [*F*(2,27) = 5.46, *p* = 0.010, *η*^2^ = 0.29]. *Post-hoc* comparisons indicated a significant difference in depression between the asymptomatic group and the symptomatic control group (*p* = 0.009). There were no significant differences found between the symptomatic experimental group and the asymptomatic group (*p* = 0.647) or symptomatic control group (*p *= 0.072). There was also a significant difference in self-reported anxiety at follow-up between groups with a large effect size [*F*(2,27) = 6.92, *p* = 0.004, *η*^2^ = 0.33]. *Post-hoc* comparisons indicated a significant difference in anxiety between the symptomatic control group and the asymptomatic group (*p* = 0.004) and symptomatic experimental group (*p* = 0.036) . No significant differences were found between the symptomatic experimental group and the asymptomatic group (*p *= 0.603). Finally, there was also a statistically significant difference in self-reported stress between groups at follow-up with a large effect size [*F*(2,27) = 7.13, *p* = 0.003, *η*^2^ = 0.35]. *Post-hoc* comparisons indicated a significant difference in stress between the symptomatic control group and the asymptomatic group (*p* = 0.003) and the symptomatic experimental group (*p* = 0.043). No differences were found between the asymptomatic group and the symptomatic experimental group (*p* = 0.499).

Within groups, the asymptomatic group reported no statistically significant changes for depression (*p* = 0.734, *η*^2^ = 0.01), anxiety (*p* = 0.656, *η*^2^ = 0.02), or stress (*p* = 0.255, *η*^2^ = 0.14) between baseline and follow-up. Similarly the symptomatic control group reported no statistically significant changes in depression (*p* = 0.834, *η*^2^ = 0.005), anxiety (*p* = 0.051, *η*^2^ = 0.36), or stress (*p* = 0.051, *η*^2^ = 0.36). However, the symptomatic experimental group, which used the mHealth application for up to 12 weeks in conjunction with CBT reported a statistically significant decrease in depression (*p* = 0.001, *η*^2^ = 0.72), anxiety (*p *= 0.015, *η*^2^ = 0.50), and stress (*p* = 0.040, *η*^2^ = 0.39) with large effect sizes.

**Figure 1:**
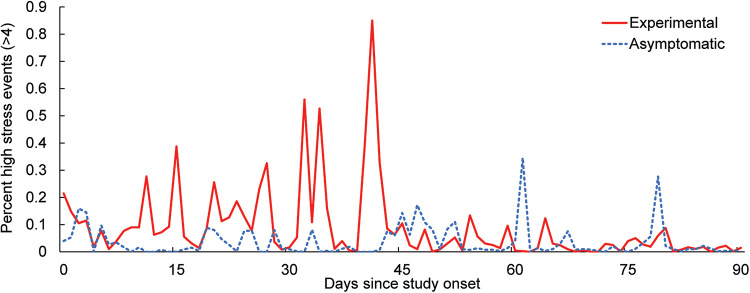
Average percent of moderate to high stress events (>4 on a 10 point scale) captured by the stress algorithm over the course of the study.

In addition to self-reported data, physiological stress calculated by the mHealth application showed a gradual decrease in moderate and high stress events (classified as >4 on a 10 point scale) as a function of time for the experimental group (Figure 1). The number of stress events in the asymptomatic group remained low throughout the study, and by approximately 45 days (6 weeks of CBT for the experimental group), the number of daily events were similar between groups.

Participants had the option of providing feedback for each stress trigger received in the application. The response rate to the stress prompts was 97.7% in the symptomatic experimental group, and 91.1% in the asymptomatic group. Across users of the mHealth system in the symptomatic experimental and asymptomatic groups, work was identified as the most common stress trigger (45.2%), followed by social stress (28.2%), other (21.8%) and anticipatory stress (4.8%). The relaxation strategies used immediately following the stress trigger was also monitored for the symptomatic experimental and asymptomatic groups. Biofeedback was the most popular strategy used (73.9%), followed by deep breathing (11.3%), progressive muscle relaxation (11.1%) and meditation (3.7%).

PROMIS-anger scores are show in Table 3. At baseline, asymptomatic participants reported none to slight anger, while both symptomatic groups reported moderate anger (23). There was a statistically significant difference between groups at baseline with a large effect size [*F*(2,27) = 14.15, *p* < 0.001, *η*^2^ = 0.51]. *Post-hoc* comparisons indicated that the asymptomatic group reported significantly less anger than the symptomatic control (*p* < 0.001) and symptomatic experimental groups (*p* = 0.002). No differences were observed between the symptomatic groups at baseline (*p *= 0.348).

**Table 3:** Mean (SD) PROMIS anger scores.

	Baseline	Follow-up
Asymptomatic (*n* = 10)	50.5 (7.1)	50.0 (9.2)
Control (*n* = 10)	69.7 (6.4)	65.6 (9.9)
Experimental (*n* = 10)	64.4 (10.8)	55.3 (9.9)^*^

* Indicates significant differences within groups at *p* ≤ 0.05.

At follow-up, there was a statistically significant difference in self-reported anger between groups with a large effect size [*F*(2,27) = 6.674, *p* = 0.004, *η*^2^ = 0.33]. *Post-hoc* comparisons indicated a significant difference in anger between the asymptomatic group and the symptomatic control group (*p* = 0.004), but not between the symptomatic experimental and asymptomatic group (*p* = 0.449) or between the experimental and control groups (*p* = 0.063). At follow-up, the experimental group's average level of anger decreased from moderate to mild but not enough to be significantly different from the symptomatic control group (23).

Within groups, a statistically significant difference in anger was observed for the symptomatic experimental group with a large effect size (*p* = 0.032, *η*^2^ = 0.42) between baseline and follow-up, but not for the asymptomatic group (*p* = 0.821, *η*^2^ = 0.006) or symptomatic control group (*p *= 0.236, *η*^2^ = 0.15).

PCL-5 scores are shown in Table 4. At the initial assessment, the asymptomatic group did not meet criteria for PTSD, while both symptomatic groups scored in a range that suggests the participants either had subthreshold PTSD or would benefit from PTSD treatment (24). There was a statistically significant difference at baseline between groups with a large effect size [*F*(2,27) = 13.10, *p* < 0.001, *η*^2^ = 0.49]. *Post-hoc* comparisons indicated that the asymptomatic group reported significantly fewer PTSD symptoms than the symptomatic control group (*p* < 0.001) and symptomatic experimental group (*p* = 0.001). No differences were observed between the two symptomatic groups (*p* = 0.823).

**Table 4:** Mean (SD) PCL-M scores.

	Baseline	Follow-up
Asymptomatic (*n* = 10)	7.2 (5.6)	8.0 (6.9)
Control (*n* = 10)	32.6 (13.3)	30.5 (15.2)
Experimental (*n* = 10)	29.4 (15.1)	20.5 (14.1)

At follow-up, there was a statistically significant difference in self-reported PTSD symptoms between groups with a large effect size [*F*(2,27) = 7.96, *p* = 0.002, *η*^2^ = 0.37]. *Post-hoc* comparisons indicated a significant difference in PTSD symptoms between the asymptomatic group and the symptomatic control group (*p* = 0.001). No significant differences were found between the symptomatic experimental group and asymptomatic group (*p* = 0.088) or symptomatic control group (*p* = 0.198).

Within groups, no difference in PTSD symptoms was observed for the asymptomatic (*p* = 0.662, *η*^2^ = 0.02), symptomatic control (*p* = 0.601, *η*^2^ = 0.03), or symptomatic experimental groups (*p* = 0.096, *η*^2^ = 0.28) between baseline and follow-up.

Epworth sleepiness scale (ESS) scores are shown in Table 5. At baseline, the asymptomatic group reported lower normal daytime sleepiness, while the symptomatic groups reported higher normal daytime sleepiness (25). There was not a statistically significant difference at baseline between groups [*F*(2,27) = 2.14, *p *= 0.138, *η*^2^ = 0.14]. At follow-up, there was a statistically significant difference in sleepiness symptoms between groups with a large effect size [*F*(2,27) = 3.84, *p* = 0.034, *η*^2^ = 0.22]. *Post-hoc* comparisons indicated a significant difference in sleepiness between the asymptomatic group and the symptomatic experimental group (*p* = 0.027). No significant differences were found between the symptomatic control group and asymptomatic group (*p* = 0.242) or experimental group (*p *= 0.521). Within groups, no difference in sleepiness was observed for the asymptomatic (*p* = 0.413, *η*^2^ = 0.08), control (*p* = 0.601, *η*^2^ = 0.031), or experimental (*p* = 0.624, *η*^2^ = 0.03) groups between baseline and follow-up.

**Table 5:** Mean (SD) epworth sleepiness scale (ESS) scores.

	Baseline	Follow-up
Asymptomatic (*n* = 10)	4.6 (2.6)	4.1 (3.5)
Control (*n* = 10)	7.5 (6.0)	7.5 (4.7)
Experimental (*n* = 10)	8.7 (4.6)	9.8 (5.6)

**Figure 2:**
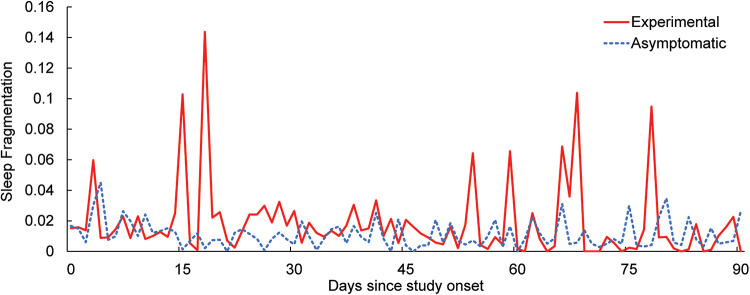
Daily average sleep fragmentation over the course of the study for the experimental and asymptomatic groups.

In addition to self-reported sleep, sleep fragmentation was calculated using movement data from the wrist-worn sensor (Figure 2). During sleep epochs, each minute is classified as “asleep” or “awake,” with fragmentation defined as a ratio of awake minutes vs. total minutes in a sleep epoch, expressed as a percentage. As is seen in Figure 2 and the self-reported data from Table 5, the symptomatic group experienced lower sleep quality throughout the study, which was not affected by the use of the mHealth application.

Participants in the asymptomatic and experimental groups rated various aspects of the mHealth application. On a scale of 1 to 5, participants rated the overall experience with the application at 3.8 ± 0.6 (SD), the user interface at 3.3 ± 1.1 (SD), the intuitiveness of the application at 4.3 ± 0.8 (SD), and the loading speed at 4.2 ± 0.9 (SD). Participants rated the stress alerts and relaxation strategies as features they liked most, and application crashes as the experience they liked least. Participants used the application on average 1.1 ± 1.2 (SD) times daily, and most participants indicated that using the mHealth application helped them to achieve their goals.

## Discussion

The current study demonstrated the feasibility of pairing data-driven mobile applications with CBT in a modest sample size in a vulnerable population, leading to an improvement in CBT compliance and a reduction in symptoms of depression, anxiety, and stress. This data is in close agreement with a similar study in a military veteran population, in which participants who used data-driven mobile applications with CBT were also significantly less likely to discontinue therapy and significantly improved on measures of stress, anxiety, and anger compared to controls undergoing CBT alone (18). Of note, all participants in the experimental and control groups were considered symptomatic for depression, anxiety, stress, anger and PTSD at baseline. At follow-up, users of the mHealth application in the experimental group reported levels of depression, anxiety, stress, anger, and PTSD that were indistinguishable from members of the asymptomatic control group, while members of the symptomatic control group retained baseline levels of depression, anxiety, stress, anger and PTSD, indicating a multiplicative effect of leveraging technology on CBT efficacy.

The symptoms targeted by the mHealth application are very common among members of the armed forces (35). For instance, severe stress was reported by nearly half of US National Guard troops (36), and a similar proportion of deployed members of the US Navy and Marines screened positive for depression (37). Anger is also a prevalent problem within the military (38). For veterans returning from the post 9/11 conflicts, problematic anger has been identified as one of the most common and pressing conditions requiring treatment (39). Survey responses from 16,699 service members showed almost 47% demonstrating significant aggressive behaviors over the past 30 days (38). Taken together, depression, stress, and anger represent highly prevalent problems in active duty service members and veterans. However, the stigma of seeking care for mental health disorders prevents the majority of military personnel from seeking help (40). Previous research has shown that data-driven mobile applications allow for the practice of CBT skills and reduction in symptoms (41), and are well-accepted by military populations (42), helping to promote effective mental health support.

Unlike previously reported CBT mobile applications (41) or laboratory applications which require expensive scientific-grade hardware to capture stress (43), the current application leveraged commercially available wrist-worn systems and the participant's personal mobile device to provide an adjunct to CBT. High accuracy real-time alerting of stress was provided through a previously reported algorithm (18) allowing participants to identify, self-assess, and reduce stress. The success of CBT depends largely on participants' compliance with practicing the coping strategies and relaxation techniques they learn in each session (15). A primary function of the mHealth system in the present study was to provide engaging resources in support of the practice occurring between sessions. Interactive alerts triggered by the stress classifier, allowed users to learn how to recognize the physiological symptoms of stress they might otherwise miss or ignore. Similarly, the in-app stress reduction tools provided users with increased awareness of their ability to reduce stress through the use of visualizations that are synced with real-time data from the sensor band. The goal was to help users gain awareness and control over physiological functions they may have otherwise missed or ignored. The current study indicated that the level of use of the mHealth system correlated with a reduction in symptoms. Such a system allows for both in-person and remote monitoring and treatment. Due to the ongoing COVID-19 pandemic, approximately half of the CBT sessions in the current study were held remotely, an approach which is becoming increasingly common across specialties (44). In addition, study therapists reported benefits from the objective data about participant stress and stress responses, and used this data to calibrate and individualize treatment more effectively and efficiently. However, therapists were not blinded to participant experimental condition, which represents a limitation in the current work.

PPG-based approaches to heart rate quantification are subject to numerous artifacts, including: movement artifacts (45); ambient light interferences, which saturates the PPG photodetectors (46); decreased reflection due to skin pigmentation (47); and alteration due to user medical state, including anemia or hypothermia (48). However, previous research has indicated that PPG-based approaches to heart rate variability assessments can be highly accurate (49), especially when contextual information is available (18). In the current study, the asymptomatic participants exhibited low stress levels on average over the course of the study. However, the number of identified stress events did vary greatly within this group. While this may be explained by individual differences in stress experienced, it is also at least partially due to the way the stress algorithm classifies suprathreshold stress events for each user. The classifier was calibrated to a baseline level for each individual. The implementation of the stress classifier in the current study required participants to manually calibrate their baseline by remaining at rest for a fixed, 5-minute period. A too-high baseline resulted in a less sensitive but more specific classifier, reducing the number of identified stress events. On the other hand, if the baseline was low, then the classifier was too sensitive, increasing the number of identified stress events and the number of false alarms. The self-baselining feature represents a limitation of the current work. Future work will include developing an algorithm that can automatically and adaptively determine the physiological baseline for each user.

The most common stressors identified by the study sample included work, social stress, other, and anticipatory stress. Military operations involve rigorous mental and physical tasks that contribute to physiological stress, altered mood, lack of sleep, and physical strain, all of which lead to high injury rates. Recent reports indicate that sleep disturbances affect a growing majority of enlisted and veteran service members (50, 51), and that thermal injury rates remain high during training and operations (52). Mission tempo, duration, frequency, altitude, and weather conditions are additional work-related stressors capable of degrading service member health and performance, and service members who experience physical or psychological injury are significantly more likely to be discharged or to resign than those who do not (53). Social interactions are considered one of the most potent human stressors (54), and were identified as the primary cause in about one third of stress responses captured in the study. Anticipatory stress, in combination with social stress, form the most potent controlled stress paradigm reported to date (55). There are many additional real-life stressors described in the literature (56), including bereavement/loss, academic examinations, anticipation of medical interventions, and public speaking, which were categorized as “other” in the current effort.

Beyond traditional approaches to human state quantification *via* body-worn or remote biosensors, machine learning is being pursued to infer meaning from the increasingly sophisticated sensors embedded in modern smartphones (57). Future work is focused on the inclusion of such passive digital phenotypes for stress, which is expected to improve user compliance and system ease of use by eliminating the need for a separate wearable device, increase data security by removing the need for wireless communication between a wearable and mobile device, and more seamlessly integrating with smartphone functions such as contacting support groups. In addition, the integration of additional data input sources, such as questionnaires, fitness testing, or other applications will expand the scope of the system beyond stress and sleep into general health and wellness. Such an aggregation is expected to provide better insights and inform decisions to improve personalized support.

## Data Availability

The raw data supporting the conclusions of this article will be made available by the authors, without undue reservation.

## References

[1] Santomauro DF Mantilla Herrera AM Shadid J Zheng P Ashbaugh C Pigott DM Global prevalence and burden of depressive and anxiety disorders in 204 countries and territories in 2020 due to the COVID-19 pandemic. Lancet. (2021) 398(10312):1700–12. 10.1016/s0140-6736(21)02143-7 34634250 PMC8500697

[2] Salari N Hosseinian-Far A Jalali R Vaisi-Raygani A Rasoulpoor S Mohammadi M Prevalence of stress, anxiety, depression among the general population during the COVID-19 pandemic: a systematic review and meta-analysis. Global Health. (2020) 16(1):57. 10.1186/s12992-020-00589-w 32631403 PMC7338126

[3] Marshall RD Olfson M Hellman F Blanco C Guardino M Struening EL. Comorbidity, impairment, and suicidality in subthreshold PTSD. Am J Psychiatry. (2001) 158(9):1467–73. 10.1176/appi.ajp.158.9.1467 11532733

[4] Yarvis J Schiess L. Subthreshold posttraumatic stress disorder (PTSD) as a predictor of depression, alcohol use, and health problems in veterans. J Workplace Behav Health. (2008) 23(4):395–424. 10.1080/15555240802547801

[5] Benincasa V Passannante M Pierrini F Carpinelli L Moccia G Marinaci T Burnout and psychological vulnerability in first responders: monitoring depersonalization and phobic anxiety during the COVID-19 pandemic. Int J Environ Res Public Health. (2022) 19(5):2794. 10.3390/ijerph19052794 35270484 PMC8910596

[6] Prasad K McLoughlin C Stillman M Poplau S Goelz E Taylor S Prevalence and correlates of stress and burnout among US healthcare workers during the COVID-19 pandemic: a national cross-sectional survey study. EClinicalMedicine. (2021) 35:100879. 10.1016/j.eclinm.2021.100879 34041456 PMC8141518

[7] Dieter JN Engel SD. Traumatic brain injury and posttraumatic stress disorder: comorbid consequences of war. Neurosci Insights. (2019) 14:1179069519892933. 10.1177/1179069519892933 32363347 PMC7176398

[8] Binns JH Barlow C Bloom FE Clauw DJ Golomb BA Graves JC Gulf War illness and the health of Gulf War veterans. Washington, DC: Dept of Veterans Affairs (2008).

[9] David D Cristea I Hofmann SG. Why cognitive behavioral therapy is the current gold standard of psychotherapy. Front Psychiatry. (2018) 9:4. 10.3389/fpsyt.2018.00004 29434552 PMC5797481

[10] Levi O Ben Yehuda A Pine DS Bar-Haim Y. A sobering Look at treatment effectiveness of military-related posttraumatic stress disorder. Clin Psychol Sci. (2021) 10(4) 690–9. 10.1177/21677026211051314

[11] Garcia HA Kelley LP Rentz TO Lee S. Pretreatment predictors of dropout from cognitive behavioral therapy for PTSD in Iraq and Afghanistan war veterans. Psychol Serv. (2011) 8(1):1–11. 10.1037/a0022705

[12] Spoont MR Nelson DB Murdoch M Rector T Sayer NA Nugent S Impact of treatment beliefs and social network encouragement on initiation of care by va service users with PTSD. Psychiatr Serv. (2014) 65(5):654–62. 10.1176/appi.ps.201200324 24488502

[13] Affairs DoV. Expanded access to non-va care through the veterans choice program. Final rule. Fed Regist. (2015) 80(209):66419–29. 26524769

[14] Davis MM Clark SJ Butchart AT Singer DC Shanley TP Gipson DS. Public participation in, and awareness about, medical research opportunities in the era of clinical and translational research. Clin Transl Sci. (2013) 6(2):88–93. 10.1111/cts.12019 23601336 PMC4743028

[15] Decker SE Kiluk BD Frankforter T Babuscio T Nich C Carroll KM. Just showing up is not enough: homework adherence and outcome in cognitive–behavioral therapy for cocaine dependence. J Consult Clin Psychol. (2016) 84(10):907. 10.1037/ccp0000126 27454780 PMC5341374

[16] Puterman E Pauly T Ruissen G Nelson B Faulkner G. Move more, move better: a narrative review of wearable technologies and their application to precision health. Health Psychol. (2021) 40(11):803–10. 10.1037/hea0001125 34855416

[17] Lee AH DiGiuseppe R. Anger and aggression treatments: a review of meta-analyses. Curr Opin Psychol. (2018) 19:65–74. 10.1016/j.copsyc.2017.04.004 29279226

[18] Winslow BD Chadderdon GL Dechmerowski SJ Jones DL Kalkstein S Greene JL Development and clinical evaluation of an mhealth application for stress management. Front Psychiatry. (2016) 7:130. 10.3389/fpsyt.2016.00130 27507949 PMC4960497

[19] Bakker D Rickard N. Engagement with a cognitive behavioural therapy Mobile phone app predicts changes in mental health and wellbeing: moodmission. Aust Psychol. (2019) 54(4):245–60. 10.1111/ap.12383

[20] Muroff J Robinson W. Tools of engagement: practical considerations for utilizing technology-based tools in cbt practice. Cogn Behav Pract. (2020) 29(1):81–96. 10.1016/j.cbpra.2020.01.004

[21] Reilly PM Shopshire MS. Anger management for substance abuse and mental health clients: a cognitive behavioral therapy manual. J Drug Addict Educ Erad. (2014) 10(1/2):199.

[22] Brown TA Chorpita BF Korotitsch W Barlow DH. Psychometric properties of the depression anxiety stress scales (dass) in clinical samples. Behav Res Ther. (1997) 35(1):79–89. 10.1016/s0005-7967(96)00068-x 9009048

[23] Pilkonis PA Choi SW Reise SP Stover AM Riley WT Cella D. Item banks for measuring emotional distress from the patient-reported outcomes measurement information system (promis®): depression, anxiety, and anger. Assessment. (2011) 18(3):263–83. 10.1177/1073191111411667 21697139 PMC3153635

[24] Weathers F Ford J. Psychometric review of PTSD Checklist (Pcl-C, Pcl-S, Pcl-M, Pcl-Pr). In: Measurement of stress, trauma, and adaptation Derwood, MD: The Sidran Press (1996). p. 250–1.

[25] Johns MW. A new method for measuring daytime sleepiness: the epworth sleepiness scale. Sleep. (1991) 14(6):540–5. 10.1093/sleep/14.6.540 1798888

[26] Garbarino M Lai M Bender D Picard RW Tognetti S, editors. Empatica E3—a wearable wireless multi-sensor device for real-time computerized biofeedback and data acquisition. Wireless Mobile Communication and Healthcare (Mobihealth), 2014 EAI 4th International Conference on; IEEE (2014).

[27] Electrophysiology TFotESoCtNASoP. Heart rate variability: standards of measurement, physiological interpretation, and clinical use. Circulation. (1996) 93(5):1043–65. 10.1161/01.CIR.93.5.1043 8598068

[28] Drigas A Mitsea E. Conscious breathing: a powerful tool for physical & neuropsychological regulation. The role of Mobile apps. Tech Soc Sci J. (2022) 28:135–58. 10.47577/tssj.v28i1.5922

[29] Minen MT Adhikari S Padikkala J Tasneem S Bagheri A Goldberg E Smartphone-Delivered progressive muscle relaxation for the treatment of migraine in primary care: a randomized controlled trial. Headache. (2020) 60(10):2232–46. 10.1111/head.14010 33200413 PMC8721526

[30] Lewis GF Hourani L Tueller S Kizakevich P Bryant S Weimer B Relaxation training assisted by heart rate variability biofeedback: implication for a military predeployment stress inoculation protocol. Psychophysiology. (2015) 52(9):1167–74. 10.1111/psyp.12455 26095854

[31] Yang E Schamber E Meyer RML Gold JI. Happier healers: randomized controlled trial of Mobile mindfulness for stress management. J Altern Complement Med. (2018) 24(5):505–13. 10.1089/acm.2015.0301 29420050

[32] Uyumaz B E Feijs L Hu J. A review of digital cognitive behavioral therapy for insomnia (cbt-I apps): are they designed for engagement? Int J Environ Res Public Health. (2021) 18(6):2929. 10.3390/ijerph18062929 33809308 PMC7999422

[33] Cohen J. Statistical power analysis for the behavioral sciences: New York, NY: Routledge (2013).

[34] Crawford JR Henry JD. The depression anxiety stress scales (dass): normative data and latent structure in a large non-clinical sample. Br J Clin Psychol (2003) 42(Pt 2):111–31. 10.1348/014466503321903544 12828802

[35] Ramchand R Rudavsky R Grant S Tanielian T Jaycox L. Prevalence of, risk factors for, and consequences of posttraumatic stress disorder and other mental health problems in military populations deployed to Iraq and Afghanistan. Curr Psychiatry Rep. (2015) 17(5):37. 10.1007/s11920-015-0575-z 25876141

[36] Khaylis A Polusny MA Erbes CR Gewirtz A Rath M. Posttraumatic stress, family adjustment, and treatment preferences among national guard soldiers deployed to oef/oif. Mil Med. (2011) 176(2):126–31. 10.7205/MILMED-D-10-00094 21366071

[37] Sharkey JM Rennix CP. Assessment of changes in mental health conditions among sailors and marines during postdeployment phase. Mil Med. (2011) 176(8):915–21. 10.7205/MILMED-D-10-00366 21882782

[38] Meadows SO Engel CC Collins RL Beckman RL Cefalu M Hawes-Dawson J 2015 Department of defense health related behaviors survey (hrbs). Rand Health Q. (2018) 8(2):5. 30323988 10.7249/rhq-08-02-05 PMC6183770

[39] Dillon KH Crawford EF Kudler H Straits-Troster KA Elbogen EB Calhoun PS. An investigation of treatment engagement among Iraq/Afghanistan era veterans with problematic anger. J Nerv Ment Dis. (2017) 205(2):119. 10.1097/NMD.0000000000000651 28098580 PMC5272832

[40] Sharp M-L Fear NT Rona RJ Wessely S Greenberg N Jones N Stigma as a barrier to seeking health care among military personnel with mental health problems. Epidemiol Rev. (2015) 37(1):144–62. 10.1093/epirev/mxu012 25595168

[41] Elbogen EB Aralis H Cassiello-Robbins CF Lester P Saltzman W Barish G. Integrating Mobile technology and social support with cognitive behavioral therapy for anger in veterans with ptsd: a pilot study. Mil Behav Health. (2021) 9(1):17–26. 10.1080/21635781.2020.1768972

[42] Morland LA Niehaus J Taft C Marx BP Menez U Mackintosh M-A. Using a Mobile application in the management of anger problems among veterans: a pilot study. Mil Med. (2016) 181(9):990–5. 10.7205/MILMED-D-15-00293 27612342

[43] Parlak O. Portable and wearable real-time stress monitoring: a critical review. Sens Actuators Rep. (2021) 3:100036. 10.1016/j.snr.2021.100036

[44] Boelen PA Eisma MC Smid GE Jd K Lenferink LI. Remotely delivered cognitive behavior therapy for disturbed grief during the COVID-19 crisis: challenges and opportunities. J Loss Trauma. (2021) 26(3):211–9. 10.1080/15325024.2020.1793547

[45] Subhagya D Aruna N Janardhan L Ramakrishna H, editors. Case study on measurement of Spo2 from Ppg signals in the presence of motion artifact. Recent Advances in Electronics and Communication Technology (ICRAECT), 2017 International Conference on; IEEE (2017).

[46] Hanowell L Eisele JH Jr. Downs D. Ambient light affects pulse oximeters. Anesthesiology. (1987) 67(5):864–5. 10.1097/00000542-198711000-00057 3674506

[47] Sinex JE. Pulse oximetry: principles and limitations. Am J Emerg Med. (1999) 17(1):59–67. 10.1016/s0735-6757(99)90019-0 9928703

[48] Chan ED Chan MM Chan MM. Pulse oximetry: understanding its basic principles facilitates appreciation of its limitations. Respir Med. (2013) 107(6):789–99. 10.1016/j.rmed.2013.02.004 23490227

[49] Lu G Yang F Taylor JA Stein JF. A comparison of photoplethysmography and ecg recording to analyse heart rate variability in healthy subjects. J Med Eng Technol. (2009) 33(8):634–41. 10.3109/03091900903150998 19848857

[50] Hughes JM Ulmer CS Gierisch JM Nicole Hastings S Howard MO. Insomnia in United States military veterans: an integrated theoretical model. Clin Psychol Rev. (2018) 59:118–25. 10.1016/j.cpr.2017.11.005 29180102 PMC5930488

[51] Moore BA Tison LM Palacios JG Peterson AL Mysliwiec V. Incidence of insomnia and obstructive sleep apnea in active duty United States military service members. Sleep. (2021) 44(7):zsab024. 10.1093/sleep/zsab024 33532830

[52] AFHSC. Update: heat injuries, active component, US armed forces, 2013. Msmr. (2014) 21(3):10. 24684615

[53] Canham-Chervak M Hauret K Hoedebecke E Laurin MJ Cuthie J. Discharges during US army basic training: injury rates and risk factors. Mil Med. (2001) 166(7):641–7. 10.1093/milmed/166.7.641 11469039

[54] Dickerson SS Kemeny ME. Acute stressors and cortisol responses: a theoretical integration and synthesis of laboratory research. Psychol Bull. (2004) 130(3):355–91. 10.1037/0033-2909.130.3.355 15122924

[55] Kirschbaum C Pirke K-M Hellhammer DH. The “trier social stress test”–a tool for investigating psychobiological stress responses in a laboratory setting. Neuropsychobiology. (1993) 28(1-2):76–81. 10.1159/000119004 8255414

[56] Biondi M Picardi A. Psychological stress and neuroendocrine function in humans: the last two decades of research. Psychother Psychosom. (1999) 68(3):114–50. 10.1159/000012323 10224513

[57] Insel TR. Digital phenotyping: technology for a new science of behavior. JAMA. (2017) 318(13):1215–6. 10.1001/jama.2017.11295 28973224

